# Barriers and facilitators for implementing active surveillance after neoadjuvant chemoradiotherapy for oesophageal cancer in Dutch hospitals: a qualitative study

**DOI:** 10.1136/bmjopen-2026-119825

**Published:** 2026-11-11

**Authors:** Sanjiv S G Gangaram Panday, Yumin Kim, Erwin Ista, Bianca Mostert, Leonieke Kranenburg, J Jan B van Lanschot, Manon C W Spaander, Jan Willem T Dekker, Joos Heisterkamp, Grard A P Nieuwenhuijzen, Jean-Pierre Pierie, Camiel Rosman, Johanna W van Sandick, Sjoerd M Lagarde, Bas P L Wijnhoven, SANO Study Group

**Affiliations:** 1 Department of Surgery, Erasmus MC Cancer Institute, Erasmus MC University Medical Centre, Rotterdam, The Netherlands; 2 Department of Internal Medicine, Division of Nursing Science, Erasmus MC University Medical Centre, Rotterdam, The Netherlands; 3 Department of Neonatal and Pediatric Intensive Care, Division of Pediatric lntensive Care, Sophia Children’s Hospital, Erasmus MC University Medical Centre, Rotterdam, The Netherlands; 4 Department of Medical Oncology, Erasmus MC Cancer Institute, Erasmus MC University Medical Centre, Rotterdam, The Netherlands; 5 Department of Psychiatry, Erasmus MC University Medical Center Rotterdam, Rotterdam, The Netherlands; 6 Department of Gastroenterology and Hepatology, Erasmus MC University Medical Centre, Rotterdam, The Netherlands; 7 Department of Surgery, Reinier de Graaf Gasthuis, Delft, The Netherlands; 8 Department of Surgery, Elisabeth Tweesteden Hospital, Tilburg, The Netherlands; 9 Department of Surgery, Catharina Hospital, Eindhoven, North Brabant, The Netherlands; 10 Department of Surgery, Frisius MC, Leeuwarden, The Netherlands; 11 Department of Surgery, Radboud University Medical Centre, Nijmegen, The Netherlands; 12 Department of Surgical Oncology, The Netherlands Cancer Institute, Amsterdam, The Netherlands

**Keywords:** Implementation Science, Oesophageal disease, Cancer

## Abstract

**Abstract:**

**Objectives:**

To explore barriers and facilitators to implementing active surveillance for patients with a complete clinical response after neoadjuvant chemoradiotherapy (nCRT) for oesophageal cancer.

**Design:**

A qualitative study using semistructured group interviews. Data were collected and analysed using the Consolidated Framework for Implementation Research (CFIR). The CFIR comprised five domains: innovation, outer setting (external healthcare environment), inner setting (hospital organisational context), characteristics of individuals and process of implementation.

**Setting:**

Six Dutch hospitals participating in the Surgery As Needed for Oesophageal cancer trial.

**Participants:**

32 medical specialists and nurse practitioners involved in active surveillance and follow-up care.

**Results:**

Barriers related to innovation included the high frequency of surveillance visits and uncertainty regarding the diagnostic value of the first clinical response evaluation (CRE) shortly after nCRT, while optimisation of the protocol (such as refining timing, frequency and criteria for CREs) was a facilitator. Facilitators in the outer setting included ongoing evidence generation through prospective studies. Within the inner setting, barriers involved challenges in coordinating follow-up planning, potential negative financial implications for surgical departments and increased workload for gastroenterology and nuclear medicine teams while existing multidisciplinary collaboration acted as a facilitator. Barriers related to characteristics of individuals included limited endoscopic experience in irradiated tissue, while patient preference for active surveillance and shared decision-making were facilitators.

**Conclusion:**

Implementation of active surveillance after nCRT for oesophageal cancer is challenged by organisational complexity and the need for ongoing generation of data on effectiveness of active surveillance. Clear allocation of responsibilities, streamlined surveillance pathways and adequate training are essential to support effective and sustainable adoption in routine clinical practice.

Trial registration number

(NTR-6803)

**STRENGTHS AND LIMITATIONS OF THIS STUDY**

- Conducted across six hospitals, enhancing the generalisability of findings to diverse clinical settings.
- Included a multidisciplinary group of participants (medical specialists and nurse practitioners), providing a broad perspective on barriers and facilitators.
- Used semistructured group interviews, allowing in-depth exploration while encouraging discussion and interaction among participants.
- Data collection and analysis were guided by the Consolidated Framework for Implementation Research (CFIR), ensuring a systematic and theory-informed approach.
- Findings may be influenced by group dynamics in interviews, potentially limiting the expression of individual views.

## Introduction

 Oesophageal cancer remains a high-impact malignancy associated with considerable morbidity and mortality.^1^ For patients with advanced locoregional disease, neoadjuvant chemoradiotherapy (nCRT) followed by surgical resection has long been the standard of care.^2^ Active surveillance can be an alternative to standard surgery for patients who achieve a complete clinical response (CCR) after nCRT. Active surveillance is defined as an organ sparing strategy where patients are only operated when cancer regrowth is detected in the absence of distant metastases. Detection of residual disease is done by repeated diagnostic tests after nCRT that include endoscopy with biopsies, endosonography with fine-needle aspiration of suspected lymph nodes and positron emission tomography (PET)-CT scan. The Surgery As Needed for Oesophageal cancer (SANO) trial showed that active surveillance is non-inferior to standard surgery in terms of 2-year overall survival.^3^ These findings suggest a potential paradigm shift from surgery in all patients towards organ preservation in selected patients after nCRT, although long-term safety and efficacy of active surveillance remain to be clarified.

The active surveillance strategy in the SANO trial required precise planning and monitoring of the diagnostic tests by the study team and restructuring of care pathways in participating centres. Therefore, the aim of the SANO-2 study, a prospective cohort study on active surveillance, was to monitor the safety of active surveillance outside the trial, with a particular focus on ensuring the correct planning of diagnostics, including the timely scheduling of clinical response evaluations (CREs) and appropriate follow-up care, to optimise patient outcomes.^4^

From both SANO studies it became clear that implementing active surveillance in routine clinical practice still poses several complexities, including planning of the diagnostic tests, interpretation of the results and rapid coordination of next steps to follow. Such intensive monitoring can strain hospital capacity including gastroenterology and radiology departments. In addition, moving from a treatment model centred on surgery for all to one focused on active surveillance may affect workflow patterns, professional responsibilities, financial flows and intensify collaboration between departments.^5^ Understanding these dynamics is essential for the successful implementation of active surveillance. This study aimed to identify barriers and facilitators to implementing active surveillance for patients with oesophageal cancer.

## Methods

We conducted a qualitative study using semistructured focus group interviews between June and September 2023 to gain an in-depth understanding of clinicians’ experiences with active surveillance who participated in the SANO trial (figure 1). The reporting of this study follows the Consolidated Criteria for Reporting Qualitative Research (COREQ) checklist.^6^

**Figure 1:**
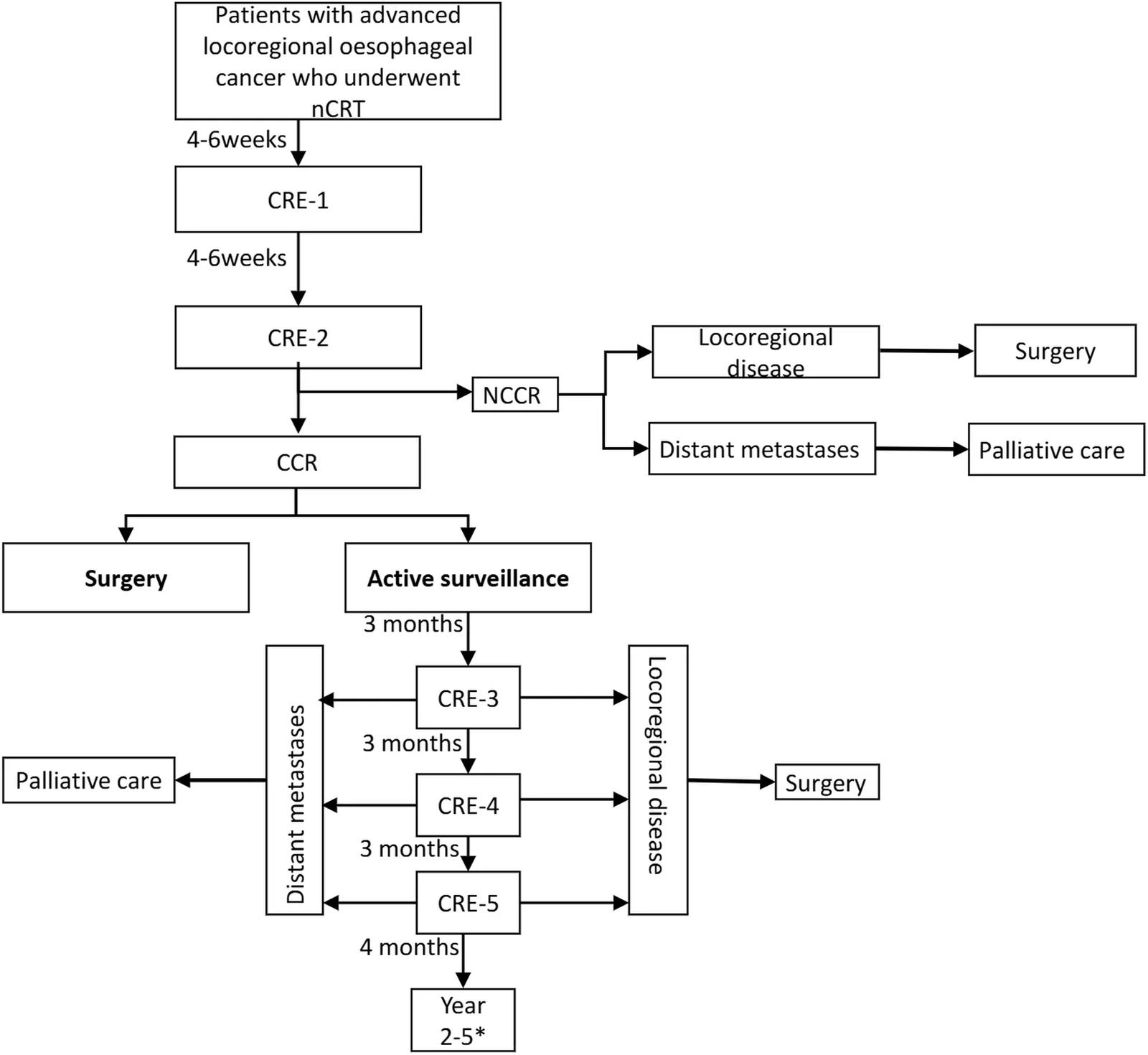
Study algorithm SANO. *Increasing interval every 4 months in the 2nd year, every 6 months in the 3rd year and yearly in year 4 and 5. Patients can also dropout/withdraw from active surveillance. CCR, complete clinical response; CRE, clinical response evaluation; NCCR, non-complete clinical response; nCRT, neoadjuvant chemoradiotherapy; SANO, Surgery As Needed for Oesophageal cancer.

### Participants

Participants were healthcare professionals from hospitals involved in active surveillance, including medical specialists and nurse practitioners from relevant disciplines: gastroenterologists, surgeons, medical oncologists, radiotherapists, nuclear medicine physicians, pathologists, research nurses and nurse practitioners. Participants were recruited (by email) from 6 (of 12) Dutch hospitals that participated in the SANO trial, including three high-volume (50 or more oesophageal resections per year) and three low-volume oesophageal cancer centres (<50). These centres varied in size, geographical location and organisational structure (one academic hospital and five teaching hospitals). Hospitals and participants were purposively selected to ensure variation in hospital type, organisational structure and professional disciplines involved in active surveillance. The number of participating centres was determined a priori to obtain representation across these settings. Data were collected through six group interviews, each including all participants from a single hospital. Participation was voluntary and dependent on clinicians’ availability on the scheduled interview dates at the hospitals. No participants withdrew during the interviews. No non-participants were present during the interviews.

### Data collection

Semistructured interviews were conducted to explore perceived barriers and facilitators related to the SANO care pathway.^7^ All interviews were conducted by SSGGP, a male first-year PhD candidate with 1 year of clinical experience as a surgical resident. SSGGP was a member of the SANO study group and involved in the SANO trial; however, he had no prior relationship with the participants. Participants were informed about his role in the study and his involvement in the SANO project before the interviews. To minimise interviewer influence, discussions were guided by a semistructured interview guide with open-ended questions and participants were encouraged to freely share their views and experiences. A predefined semistructured interview guide was developed by the SANO study research group and structured according to the Consolidated Framework for Implementation Research (CFIR) (online supplemental text S1).^8^ The CFIR comprises five domains: innovation characteristics (the study protocol and care pathway), outer setting (patient organisations, health insurers and non-participating hospitals as stakeholders), inner setting (hospital departments and multidisciplinary collaborations within centres), characteristics of individuals (including physicians, nurse practitioners and research nurses) and the process of implementation. Insights from each group interview were used to inform and refine the topic guide for subsequent group interviews. Following the fourth interview, no further changes to the interview guide were required and no substantially new information emerged in the subsequent interviews. Although formal data saturation was not assessed, these observations suggested that the included sample provided sufficient information to address the study aim. The interviews primarily focused on the first four CFIR domains, as the implementation process domain becomes relevant after identification of barriers and facilitators. Although participants consisted solely of healthcare professionals, perspectives on all domains were explored to obtain a comprehensive overview of implementation determinants from a clinical viewpoint.

Each interview started with a brief introduction to CFIR and implementation science concepts. This was followed by open-ended questions that increasingly focused on more specific topics. Interviews explored experiences with the SANO care pathway, perceived challenges, organisational context and perspectives on nationwide implementation. Each interview was planned for 60 min but the duration depended on participant input. All interviews were audio-recorded, and transcribed verbatim by SSGGP to ensure an accurate record of participants’ responses.

### Data analysis

Interview data were analysed in ATLAS.ti (ATLAS.ti Scientific Software Development GmbH, Berlin, Germany) using direct content analysis with multiple steps. The initial codebook was guided by the CFIR-based topic list. The analysis proceeded through several steps: First, the transcripts were coded by a researcher (YK) using open codes and all codes were categorised within the four CFIR domains. Second, all codes were checked by another researcher (SSGGP). Codes were then grouped under the predefined topics, and additional topics were added when appropriate. Next, all extracted content was compared and discussed by YK and SSGGP until consensus was reached. If consensus could not be reached, a third researcher (BPLW) was consulted. Themes were reviewed and refined as necessary to ensure the results were presented coherently. Representative quotes were selected to illustrate themes (paraphrased to ensure anonymity).

To enhance trustworthiness, we employed a systematic and transparent approach to data collection and analysis. Analytical decisions were documented throughout the study to promote transparency and consistency. Researchers with different levels of involvement in the SANO trial contributed to the analysis: YK was not involved in the SANO trial, whereas SSGGP and BPLW were members of the SANO study group. The involvement of both independent and SANO-affiliated researchers facilitated critical reflection on data interpretation and helped minimise the influence of individual perspectives. Coding discrepancies and interpretations were discussed until consensus was reached, with consultation of a third researcher (BPLW) when required. Participants did not review transcripts or study findings (member checking was not performed).

### Patient and public involvement

A patient representative from De Stichting SPKS – Leven met maag- of slokdarmkanker was actively involved in the design of this study. They provided input on the study objectives, interview topics and phrasing of questions to ensure relevance and clarity from the patient perspective. Their feedback helped to align the study with patient priorities and enhanced the practical applicability of the findings.

## Results

In total, 42 clinicians attended the six semistructured focus group interviews, of whom 32 actively contributed during the sessions. Relevant medical specialties were all represented (table 1). Interviews lasted a mean of 48 min (range 39–57 min). Identified determinants, including both barriers and facilitators, were thematically organised within the four CFIR domains, with 13 determinants identified (table 2). Across interviews, determinants were largely consistent across professional disciplines. Where variation occurred, this was primarily related to local organisational structures and workflows rather than specialty-specific perspectives.

**Table 1:** Participant specialties per semistructured group interview

Healthcare professional	Participating hospital						Total
	A	B	C	D	E	F	
Gastroenterologist	1	1	2	1	1	1	7
Oncologist	1	1	1	2	1	1	7
Pathologist	1	1	1	1	–	–	4
Radiation oncologist	1	1	–	3	1	–	6
Radiologist or nuclear medicine physician	–	–	1	–	1	1	3
Surgeon	–	1	1	1	–	3	6
Research nurse or nurse practitioner	1	1	2	1	2	2	9
Total participants	5	6	8	9	6	8	42*

* Of the 42 attendees, 32 actively contributed to the discussions and were included in the analysis.

**Table 2:** Thematical organisation of identified barriers and facilitators for implementation of the SANO protocol

CFIR domain	Theme	Type
Innovation		
	Perceived diagnostic intensity and surveillance frequency	–
	The clinical value of CRE-1	±
	Endoscopic ultrasound on indication of positive lymph nodes on PET-CT	+
Outer setting		
	Involvement of national working groups	+
	Ongoing evidence generation	+
Inner setting		
	Planning burden and coordination challenges	–
	Increased workload for gastroenterology and nuclear medicine teams	–
	Potential negative financial implications for surgical departments	–
	Multidisciplinary collaboration	+
	Lead clinician/case manager	±
Characteristics of individuals		
	Limited endoscopic experience in irradiated tissue	–
	Patient preference as perceived by clinicians	+

(+) facilitator; (–) barrier; (±) mixed perspectives, with participants describing both barrier and facilitator aspects of the determinant.

CFIR, Consolidated Framework for Implementation Research; CRE, clinical response evaluation; PET, positron emission tomography; SANO, Surgery As Needed for Oesophageal cancer.

### Innovation

#### Perceived diagnostic intensity and surveillance frequency

The SANO surveillance protocol dictates regular diagnostic tests after nCRT. Nurses from the centres described the short time span between nCRT, endoscopy (CRE-1) and CRE-2 as a limitation, noting consequences for both patient’s well-being/experience and feasibility of planning. As one nurse practitioner stated, “Planning is difficult because it is close to CRE-1”. Gastroenterologists similarly reported limited diagnostic capacity, particularly when the order of PET-CT first followed by endoscopy is required. Although these concerns were not universally expressed across all hospitals, participants shared unease about whether the surveillance protocol, in its current form, can be readily integrated into routine clinical practice.

#### The clinical value of CRE-1

There was discussion on the clinical relevance of doing a CRE-1. Several clinicians questioned whether CRE-1 meaningfully altered management decisions. One gastroenterologist noted, “It might make more sense to take the biopsy later in time, because the likelihood of a reliable result is higher”. Another gastroenterologist echoed similar concerns, raising doubts about endoscopic accuracy at such an early time point post-nCRT: “The less hassle for the patient, the better”. Other participants offered reflections on what they deemed to be the importance of CRE-1. They suggested that CRE-1 might help identify clear non-responders or progressive disease but lacked discriminative value for refining the patient’s longer-term trajectory. One radiotherapist commented “What you definitely do not want is to let patients wait longer for curative surgery when there is no response to chemoradiotherapy”. Another gastroenterologist stressed the advantage of having performed CRE-1 before CRE-2: “With CRE-2, I can follow the healing process properly; the timing helps me to assess the response”.

**Endoscopic ultrasound (EUS) on indication**

EUS was frequently described as a procedure of which the routine role requires reconsideration. Many clinicians favoured an on-indication approach, which could be a facilitator. One gastroenterologist explained, “You could also choose to perform it only for positive lymph nodes on PET-CT… that makes planning easier”. Another added, “If you see nothing on the PET-CT, does EUS still add value? That would be the question”. Multiple participants emphasised the need for more empirical data before reducing EUS frequency.

### Outer setting

#### National working groups, and ongoing evidence generation

Clinicians emphasised the importance of national working groups and guidelines in supporting implementation and standardising active surveillance across centres. These structures were seen as essential for aligning practice, sharing experience and ensuring consistency in patient care. One nurse practitioner said, “If there is no longer a protocol and restaging becomes the standard guideline, offering and planning active surveillance becomes straightforward, as it is integrated into routine care”. Clinicians also highlighted the need for collecting evidence confirming the efficacy and safety of active surveillance. It was noted that broad implementation would require reassurance that patient outcomes are not compromised. The SANO-2 study, providing a prospective cohort with monitored safety, was viewed positively as it allows hospitals to gain experience with active surveillance diagnostics while contributing to the evidence base.

### Inner setting

#### Planning burden and coordination challenges

Planning active surveillance was the most frequently mentioned challenge within the inner-setting domain. Clinicians across five of six hospitals described scheduling appointments as complex. There were differences between centres. One research nurse described a highly structured system: “Case managers ensure that in the patient record it is clear when appointments should be scheduled over the next five years…for us, that works perfectly”. By contrast, another research nurse noted, “Many departments are involved…if no one person keeps track, it becomes quite difficult” . Personal ownership of the workflow was viewed as beneficial for continuity and accuracy.

#### Redistribution of workload

The shift in workload toward gastroenterology and nuclear medicine was mentioned as a challenge. Gastroenterologists reported increases in complex endoscopies. One gastroenterologist explained, “Logistically, we are struggling; we see that we need to progressively deliver more endoscopy capacity, and that is really a problem”. In contrast, surgeons discussed concern about maintaining sufficient surgical case volume.

#### Multidisciplinary collaboration

Despite operational challenges, multidisciplinary meetings consistently emerged as one of the strongest facilitators for implementing the SANO approach. Clinicians across five of six hospitals described these meetings as essential to share uncertainty, align differing interpretations and reinforce collective decision-making. However, the extent to which these meetings supported implementation varied between centres. One medical oncologist highlighted that preoperative cases are not discussed to avoid overloading the meetings: “We only discuss patients after surgery, not beforehand” . In contrast, clinicians at another hospital emphasised the strength of their integrated multidisciplinary hospital structure: “We have a multidisciplinary clinic… there we discuss the different treatment options”. Across centres, multidisciplinary meetings were widely experienced as stabilising and clarifying, and essential for navigating complex surveillance decisions, even if involvement and timing differed.

#### Lead clinician

Clinicians expressed uncertainty about who should serve as the primary clinician during different stages of care. Some centres assigned this role to surgeons, others to gastroenterologists or medical oncologists. A surgeon noted, “If the patient still has disease… it is helpful if they are seen immediately by a surgeon”. A medical oncologist described variation depending on the situation: “With us… the gastroenterologist or medical oncologist is the primary clinician”. In another hospital, a surgeon emphasised responsibility transitions during nCRT: “The gastroenterologist is the primary clinician until diagnostics are complete… after that, it is the surgeon”. This variation reflects differences in practice across centres.

### Characteristics of individuals

#### Experience with endoscopy

Gastroenterologists across five of six hospitals reported a mixed experience with visual assessment in irradiated tissue. They emphasised that performing high-quality endoscopy after chemoradiotherapy is technically demanding, particularly during early response evaluations. Many described the postradiation mucosa as unpredictable, making it difficult to distinguish treatment effect from residual disease.

Clinicians at some centres noted that only a few endoscopists perform these examinations as part of the SANO protocol, concentrating expertise but creating vulnerability to capacity shortages. One clinician explained, “It always ends up with one of the two of us”. In contrast, other clinicians reported a more routine experience, with several endoscopists performing assessments and accustomed to upper gastrointestinal oncology. One clinician commented, “I do not feel that this is a barrier for us, neither for planning nor for performing the endoscopies”. Overall, most described a narrow pool of skilled endoscopists and intrinsic diagnostic uncertainty in irradiated tissue as important challenges.

#### Patient preference as perceived by clinicians

Clinicians across centres reported that many patients express an intrinsic preference for conservative management (or active surveillance). Physicians emphasised the importance of repeated opportunities to explain options and support patient decision-making. One nurse practitioner noted, “I have the impression many patients bring it (conservative treatment) up themselves”. Facilitators included structured shared decision-making during multiple outpatient clinical visits.

## Discussion

This qualitative study explored clinicians’ perspective on barriers and facilitators for implementing active surveillance after nCRT in patients with CCR for oesophageal cancer. Across six Dutch hospitals, clinicians from multiple specialties described a complex interplay between logistical demands, diagnostic uncertainty and increased workload as barriers for implementing active surveillance. However, ongoing evidence generation was seen as critical for confirming the efficacy of active surveillance and facilitates multidisciplinary collaboration and patient involvement. This is the first study to systematically identify implementation determinants of active surveillance after nCRT for oesophageal cancer using the CFIR framework, providing a foundation for targeted implementation strategies. Overall, implementation challenges appeared to be driven primarily by organisational and logistical complexity rather than by consistent differences between professional groups, highlighting the importance of organisational readiness for successful implementation.

Our findings were in line with comparable active surveillance practices such as in rectal cancer. The implementation of watch-and-wait therapy in rectal cancer was accompanied by similar challenges, such as logistical complexity and the need for reliable diagnostics.^5^ Despite being based on non-randomised data, watch-and-wait has gained attractivity in rectal cancer treatment through large patient datasets that demonstrated its feasibility and benefits in preserving quality of life.^9 10^ Much like in oesophageal cancer, the adoption of this strategy requires addressing policy barriers, standardising protocols and ensuring access to specialised resources. These challenges, combined with the need for ongoing evidence generation and multidisciplinary collaboration, highlight the complexities involved in shifting to a surveillance-focused model in both cancers. Similar implementation challenges have been reported in active surveillance for prostate cancer, particularly regarding communication, care coordination and healthcare processes.^11^ However, for oesophageal cancer, the SANO trial provides evidence in a randomised non-inferiority setting, offering a more robust foundation for the adoption of active surveillance in a select group of patients.^3^

The frequency of CREs in the first 2 years following nCRT is viewed as essential for the timely detection of residual tumours, allowing patients to be safely treated within the SANO trial. While CRE-1 may miss some residual tumours, it detects early abnormalities in approximately one-third of patients, despite the challenge of distinguishing treatment effects from residual disease. There is room for optimisation in the use of EUS, which could shift from a routine to an indication-based approach during later surveillance phases, as our study demonstrated it was safe to omit 97.8% of EUS procedures. EUS would only be performed if PET-CT indicates suspicious findings and only 2.7% of patients will show Fluorodeoxyglucose-avid lymph nodes.^12^ This change would alleviate the workload on gastroenterology departments, as only select patients would need EUS. With protocol adjustments, the order of tests could be modified, allowing endoscopy and PET-CT to be performed on the same day, with separate EUS appointments when needed. For a minority of patients, this may result in an additional endoscopy appointment; however, the overall benefit would be a more streamlined and efficient surveillance process, with many patients having all diagnostic tests on a single day rather than two.

Although this study focused on healthcare professionals, clinicians noted that many patients express a preference for an active surveillance approach. Repeated outpatient clinical visits facilitated shared decision-making on trial participation. However, patient views can significantly impact implementation, meaning that patient-related factors should be considered separately from organisational or clinician factors.^13^ During the writing of the SANO protocol, the Dutch patient organisation for patients with oesophagogastric cancer (SPKS) was involved and was updated on relevant outcomes during the progress of the study, ensuring patient input was incorporated into the development of the treatment strategy. Previous studies have already assessed patient’s willingness to opt for an active surveillance strategy, the need for decision counselling and what trade-offs patients are willing to make in terms of overall survival to preserve their oesophagus.^14–17^ Future research should focus on patient perspectives through focus group interviews to explore their view on the long-term outcomes, advantages and disadvantages when these long-term outcomes become available at the end of 2026. Additionally, ongoing studies on active surveillance such as the German ESORES trial and the forthcoming Chinese SINO trial will determine the acceptance of active surveillance worldwide.^18 19^ A financial re-evaluation and the development of a healthcare product for active surveillance seem necessary, given the differences in the care pathway. Ongoing discussions with insurance providers present an opportunity to ensure that the transition to a surveillance-based model remains financially sustainable for all stakeholders. Results from the cost-effectiveness analysis of the SANO-trial demonstrate the benefits of active surveillance, which should facilitate these discussions and make the transition smoother.^20^ However, successful implementation will also require alignment of financial incentives with the surveillance care pathway. While active surveillance may reduce surgical volume, it increases demands on gastroenterology, nuclear medicine, coordination and long-term follow-up services. Appropriate reimbursement structures are therefore essential to ensure that implementation remains financially sustainable across specialties and healthcare organisations.

As the structure and responsibilities for coordination vary across hospitals, one of the most important barriers or facilitators is the presence/absence of a case manager or nurse practitioner who oversees patients’ care paths. The frequent diagnostic tests and the order in which they should be done, including reporting test results to patients, require robust coordination. Multidisciplinary meetings are crucial, especially in cases of uncertain test outcomes and decision making on when to operate or not requires consensus between different specialties.

Performing endoscopy with bite-on-bite biopsies using narrow band imaging in the irradiated oesophagus is safe. The efficacy, however, depends on the clinician’s expertise.^21^ This highlights the importance of expertise and protected endoscopy capacity, particularly during early response assessments. Additionally, the process could be simplified, as taking more biopsies may be more effective than attempting bite-on-bite biopsies.^22^ If EUS is performed based on indications, more gastroenterologists could be eligible to perform the initial endoscopic procedure of the CRE, consisting of endoscopy, as they do not need to be trained in EUS to qualify.

This study provides valuable insights into the barriers and facilitators of implementing active surveillance after nCRT for oesophageal cancer, based on perspectives from a broad range of clinicians across multiple centres. The use of the CFIR framework provides a structured approach, and the involvement of diverse healthcare professionals enhances the comprehensiveness of the findings. As a qualitative study, the findings are not generalisable to all healthcare settings and rely on the experiences of enthusiastic clinicians from six hospitals. The interviews were driven by specific questions from the formulated topic list developed by the SANO study group, which may limit the scope of perspectives. However, the interviews were started with open-ended questions to allow for a broader range of responses. Furthermore, SSGGP and BPLW were involved in the SANO trial, which may have influenced data collection and interpretation. To mitigate this risk, interviews were conducted using a predefined semistructured interview guide with open-ended questions, and initial coding was performed by an independent researcher (YK) who was not involved in the SANO trial. Formal data saturation was not assessed, although no substantially new information emerged during the final interviews. As group interviews were used, some participants may have been less inclined to express dissenting opinions in the presence of colleagues. Additionally, patient perspectives were not directly captured, limiting the understanding of how patient preferences influence the implementation of active surveillance. Furthermore, while the evidence for active surveillance after nCRT is promising, the long-term impact and broader applicability need further validation.

## Conclusion

The findings on barriers and facilitators presented in this article can assist in the development of implementation strategies aimed at optimising care in patients with oesophageal cancer. Several taxonomies and methods are helpful to align identified barriers and facilitators with the specific implementation strategies.^23^ We identified important implementation challenges, including logistical demands, diagnostic uncertainty and increased workload. Our findings suggest that successful implementation of active surveillance requires organisational readiness, characterised by coordinated follow-up pathways, clear allocation of responsibilities, multidisciplinary collaboration and specialised expertise. For policymakers, sustainable reimbursement structures, national guidance and continued evidence generation may support broader implementation. While active surveillance was generally considered feasible, sustained evidence on its efficacy and safety remains essential for widespread adoption.

## Supplementary material

10.1136/bmjopen-2026-119825 online supplemental file 1

## Data Availability

Data are available upon reasonable request.

## References

[1] Low DE, Kuppusamy MK, Alderson D (2019). Benchmarking Complications Associated with Esophagectomy. Ann Surg.

[2] Eyck BM, van Lanschot JJB, Hulshof MCCM (2021). Ten-Year Outcome of Neoadjuvant Chemoradiotherapy Plus Surgery for Esophageal Cancer: The Randomized Controlled CROSS Trial. J Clin Oncol.

[3] van der Wilk BJ, Eyck BM, Wijnhoven BPL (2025). Neoadjuvant chemoradiotherapy followed by active surveillance versus standard surgery for oesophageal cancer (SANO trial): a multicentre, stepped-wedge, cluster-randomised, non-inferiority, phase 3 trial. Lancet Oncol.

[4] van der Zijden CJ, Lagarde SM, Hermus M (2023). A prospective cohort study on active surveillance after neoadjuvant chemoradiotherapy for esophageal cancer: protocol of Surgery As Needed for Oesophageal cancer-2. BMC Cancer.

[5] Loria A, Ramsdale EE, Aquina CT (2024). From Clinical Trials to Practice: Anticipating and Overcoming Challenges in Implementing Watch-and-Wait for Rectal Cancer. JCO.

[6] Tong A, Sainsbury P, Craig J (2007). Consolidated criteria for reporting qualitative research (COREQ): a 32-item checklist for interviews and focus groups. Int J Qual Health Care.

[7] Noordman BJ, Wijnhoven BPL, Lagarde SM (2018). Neoadjuvant chemoradiotherapy plus surgery versus active surveillance for oesophageal cancer: a stepped-wedge cluster randomised trial. BMC Cancer.

[8] Damschroder LJ, Aron DC, Keith RE (2009). Fostering implementation of health services research findings into practice: a consolidated framework for advancing implementation science. Implementation Sci.

[9] Habr-Gama A, Perez RO, Nadalin W (2004). Operative Versus Nonoperative Treatment for Stage 0 Distal Rectal Cancer Following Chemoradiation Therapy. Ann Surg.

[10] van der Valk MJM, Hilling DE, Bastiaannet E (2018). Long-term outcomes of clinical complete responders after neoadjuvant treatment for rectal cancer in the International Watch & Wait Database (IWWD): an international multicentre registry study. Lancet.

[11] Chen JC, Subramanian L, Skolarus TA (2025). Multi-level Factors to Build Confidence and Support in Active Surveillance for Low-Risk Prostate Cancer: A Qualitative Study. J Gen Intern Med.

[12] Gangaram Panday SSG, Pittacolo M, Lagarde SM (2026). The value of routine endoscopic ultrasound in patients with esophageal cancer undergoing active surveillance after neoadjuvant chemoradiotherapy. Endoscopy.

[13] Kane HL, Halpern MT, Squiers LB (2014). Implementing and evaluating shared decision making in oncology practice. CA Cancer J Clin.

[14] Hermus M, van der Wilk BJ, Chang R (2023). Esophageal cancer patients’ need for information and support in making a treatment decision between standard surgery and active surveillance. Cancer Med.

[15] Hermus M, van der Wilk BJ, Chang RTH (2023). Patient preferences for active surveillance vs standard surgery after neoadjuvant chemoradiotherapy in oesophageal cancer treatment: The NOSANO-study. Int J Cancer.

[16] Hermus M, van der Zijden CJ, Wijnhoven BPL (2024). Patients’ Preferences Towards Decision Counseling for Active Surveillance After Neoadjuvant Chemoradiotherapy for Esophageal Cancer. Ann Surg Oncol.

[17] Noordman BJ, de Bekker-Grob EW, Coene PPLO (2018). Patients’ preferences for treatment after neoadjuvant chemoradiotherapy for oesophageal cancer. Br J Surg.

[18] Hipp J, Bruns CJ, Schmoor C (2024). ESORES-Trial: Surgery as needed versus surgery on principle in patients with postneoadjuvant clinical complete tumor response of esophageal cancer. JCO.

[19] Yang Y, Liu Z, Wong I (2025). Detecting residual disease after neoadjuvant chemoradiotherapy for oesophageal squamous cell carcinoma: The prospective multicentre preSINO trial. *Br J Surg*.

[20] Gangaram Panday SSG, van Klaveren D, Lagarde SM (2026). Surveillance vs Standard Surgery and Cost-Effectiveness After Neoadjuvant Chemoradiotherapy for Esophageal Cancer: Secondary Analysis of a Randomized Clinical Trial. *JAMA Netw Open*.

[21] Ng SC, Lau JYW (2011). Narrow-band imaging in the colon: limitations and potentials. J Gastroenterol Hepatol.

[22] van der Bogt RD, van der Wilk BJ, Oudijk L (2022). Bite-on-bite biopsies for the detection of residual esophageal cancer after neoadjuvant chemoradiotherapy. Endoscopy.

[23] Fernandez ME, Ten Hoor GA, van Lieshout S (2019). Implementation Mapping: Using Intervention Mapping to Develop Implementation Strategies. Front Public Health.

