# Supplementary material for: Barriers and facilitators for implementing active surveillance after neoadjuvant chemoradiotherapy for oesophageal cancer in Dutch hospitals: a qualitative study
Source: BMJ Open. 2026 Nov 11;16(11):e119825. doi: 10.1136/bmjopen-2026-119825 (PMC13756475; doi:10.1136/bmjopen-2026-119825)
Supplement: Supplementary file 1 [file bmjopen-2026-119825.reviewer_comments.pdf]

PEER REVIEW HISTORY

BMJ Open publishes all reviews undertaken for accepted manuscripts. Reviewers are asked to complete a checklist review form and are provided with free text boxes to elaborate on their assessment. These free text comments are reproduced below.

ARTICLE DETAILS

Title (Provisional)

Barriers and Facilitators for implementing active surveillance after neoadjuvant chemoradiotherapy for oesophageal cancer in Dutch hospitals: a qualitative study

Authors

Gangaram Panday, Sanjiv S.G.; Kim, Yumin; Ista, Erwin; Mostert, Bianca; Kranenburg, Leonieke; van Lanschot, J. Jan B.; Spaander, Manon C.W.; Dekker, Jan Willem T.; Heisterkamp, Joos; Nieuwenhuijzen, Grard A.P.; Pierie, Jean-Pierre; Rosman, Camiel; van Sandick, Johanna W.; Lagarde, Sjoerd M.; Wijnhoven, Bas P.L.; SANO Study Group, -

---

VERSION 1 - REVIEW

**Reviewer**	**1**
**Name**	**Hamamoto, Yasuo**
**Affiliation**	**Keio University**
**Date**	**07-May-2026**
**COI**	**NA**

This is an interesting qualitative study exploring barriers and facilitators to implementing active surveillance after nCRT for oesophageal cancer using the CFIR framework. The topic is clinically relevant, particularly in light of the paradigm shift suggested by the SANO trial.

However, several important methodological and interpretative issues limit the clarity, reproducibility, and scientific rigor of the manuscript. Major revision is required to improve transparency of the qualitative methodology and to strengthen the interpretation of the findings.

Major comments

1. Lack of methodological clarity and transparency

The description of the qualitative methodology is insufficient and raises several concerns. It is unclear whether this approach follows an established qualitative methodology or represents an ad hoc collection of opinions.

The authors state that semi-structured interviews were conducted, but the details are insufficient:

Were the interviews based on a predefined interview guide? (only briefly mentioned in supplementary material)

Were all sessions audio-recorded and transcribed verbatim for all participants?

How were data saturation and sample size determined?

How many interviewers were involved, and what were their backgrounds?

Were interviews conducted by independent researchers or clinicians involved in the SANO trial?

In addition, the process of data summarisation and theme generation is not sufficiently detailed. More explicit description of coding procedures, reflexivity, and strategies to ensure trustworthiness (e.g., triangulation, member checking) is needed.

2. Interpretation limited to descriptive aggregation

The current results largely resemble a collection of opinions rather than a structured synthesis. The manuscript would benefit from a clearer integration of findings across institutions and disciplines.

It remains unclear whether there were conflicting opinions between specialties (e.g., surgeons vs gastroenterologists) and how these differences were handled.

The authors should provide a more analytical interpretation beyond listing barriers and facilitators.

Without deeper synthesis, the study risks being perceived as a descriptive survey rather than a rigorous qualitative analysis.

3. Positioning within existing literature

The discussion of similar qualitative or implementation studies is limited.

Are similar CFIR-based or qualitative studies available in this field (e.g., other cancers or surveillance strategies)?

How does this study add to existing implementation science literature?

Providing a clearer context would help readers understand whether this approach is standard and how generalisable the findings are.

4. Tables are difficult to interpret

The tables (particularly Table 2) are difficult to interpret.

The meaning of symbols (e.g., - / + / -+) is not sufficiently explained.

The table structure is overly condensed and lacks clarity.

It is difficult to understand the relative importance or frequency of each theme.

The tables should be revised to improve readability and interpretability, possibly by adding explanations, examples, or quantification where appropriate.

5. Need for clearer synthesis and take-home message

The manuscript would benefit from a more structured synthesis of findings.

A conceptual model or summary figure integrating barriers and facilitators would enhance clarity.

Practical implications for clinicians and policymakers should be more explicitly stated.

Currently, the conclusions remain somewhat generic and do not fully reflect the richness of the data.

**Reviewer**	**2**
**Name**	**Prasad, Pooja**
**Affiliation**	**Royal Victoria Infirmary**
**Date**	**20-Jun-2026**
**COI**	**NA**

A well-written article addressing a relevant issue given the increasing focus on organ-preservation strategies in this patient population. Appropriate study question and methods to address this. Clear that capturing patient/participant experience is vital and will need to be captured at the end of the prospective study. A few areas that will need to be addressed when revised:

1. No. of clinicians- 42 attended and 32 contributed. Are there reasons for 10 participants not providing data or for this to not be included?

2. Data saturation- was this reached? Did the iterative refinement of the topic guide after each set of interviews suggest data saturation was being reached? This is important w regards to qualitative studies and should be addressed

3. Limitations- reflexivity ought to be discussed given it is a component of the COREQ checklist used and there is potential for bias with regards to whether limitations/barriers can be captured accurately

4. Financial implications- key barrier to implementation recognised and mentioned but the discussion lacks adequate exploration of this

Few minor formatting / grammatical issues to address:

1. Table 2- key for facilitators/barriers symbol and needs clear formatting (+/- for example)

2. Page 9 Line 26- 'practitioner' --> incorrect spelling (manuscript says practicioner)

3. reference 11- no DOI etc included. If this is unpublished data or in press, please state this explicitly (given it is the same study group and addressing the topic of EUS utility in surveillance strategies)

**Reviewer**	**3**
**Name**	**Mughal, Muntzer**
**Affiliation**	**University College London Hospitals NHS Foundation Trust**
**Date**	**20-Aug-2026**
**COI**	**NA**

This is an excellent study to prepare the broad healthcare ecosystem for the likely incorporation of active surveillance after neoadjuvant chemoradiotherapy for oesophageal cancer.

The abstract needs to explain the acronyms as well as terminology specific to this study: 'inner setting' and 'outer setting'. Otherwise, the purpose of the abstract is lost, considering that many readers look at the abstract to, in my opinion, justifiably decide whether to read the entire paper.

VERSION 1 - AUTHOR RESPONSE

Reviewer: 1
Dr. Yasuo Hamamoto, Keio University
Comments to the Author:
This is an interesting qualitative study exploring barriers and facilitators to implementing active surveillance after nCRT for oesophageal cancer using the CFIR framework. The topic is clinically relevant, particularly in light of the paradigm shift suggested by the SANO trial.

However, several important methodological and interpretative issues limit the clarity, reproducibility, and scientific rigor of the manuscript. Major revision is required to improve transparency of the qualitative methodology and to strengthen the interpretation of the findings.

Response:
We thank the reviewer for the careful evaluation of our manuscript and for the constructive comments. We appreciate the positive assessment of the clinical relevance of this study and agree that additional methodological detail would improve the transparency and reproducibility of the work. In response to the reviewer's suggestions, we have revised the

manuscript to provide a more comprehensive description of the qualitative methodology, including the development and use of the interview guide, data collection procedures, and analytical approach. We have also clarified several aspects of the interpretation of our findings to strengthen the overall rigor of the manuscript. Detailed responses to each comment are provided below.

Major comments
1. Lack of methodological clarity and transparency

The description of the qualitative methodology is insufficient and raises several concerns. It is unclear whether this approach follows an established qualitative methodology or represents an ad hoc collection of opinions.
The authors state that semi-structured interviews were conducted, but the details are insufficient:

Were the interviews based on a predefined interview guide? (only briefly mentioned in supplementary material)

Response:
Thank you for this comment. The interviews were based on a predefined semi-structured interview guide developed by the SANO study research group and structured according to the CFIR framework. To make this clearer, we have revised the Methods section accordingly and continue to provide the complete interview guide in Supplementary Text S1.

"A predefined semi-structured interview guide….."

Were all sessions audio-recorded and transcribed verbatim for all participants?

Response: All group interviews were audio-recorded and transcribed verbatim, as stated in the Methods section:

"All interviews were audio-recorded and transcribed verbatim by SGP to ensure an accurate record of participants' responses."

Therefore, no changes were made to the manuscript.

How were data saturation and sample size determined?

Response: Hospitals and participants were purposively selected to ensure variation in hospital type, organisational structure, and professional disciplines involved in active surveillance. The number of participating centres was determined a priori to obtain representation across these settings. Formal data saturation was not used to determine the

sample size, as participating hospitals were selected a priori to ensure representation of different hospital types and disciplines. However, following the fourth interview, no further changes to the interview guide were required and no substantially new information emerged in the subsequent interviews. We have clarified this in the Methods section.

“Hospitals and participants were purposively selected to ensure variation in hospital type, organisational structure, and the professional disciplines involved in active surveillance. The number of participating centres was determined a priori to obtain representation across these settings. Based on the six participating hospitals, we expected that this number to be sufficient to achieve data saturation.”

“Following the fourth interview, no further changes to the interview guide were required and no substantially new information emerged in the subsequent interviews. Although formal data saturation was not assessed, these observations suggested that the included sample provided sufficient information to address the study aim.”

How many interviewers were involved, and what were their backgrounds?
Were interviews conducted by independent researchers or clinicians involved in the SANO trial?

Response: All interviews were conducted by a single interviewer (SGP), a researcher involved in the SANO trial. To improve transparency, we have added information on the interviewer's role. We have also clarified that a semi-structured interview guide with open-ended questions was used to encourage participants to freely express their views and experiences.

“All interviews were conducted by SGP, a researcher involved in the SANO trial. To minimise interviewer influence, discussions were guided by a semi-structured interview guide with open-ended questions and participants were encouraged to freely share their views and experiences.”

In addition, the process of data summarisation and theme generation is not sufficiently detailed. More explicit description of coding procedures, reflexivity, and strategies to ensure trustworthiness (e.g., triangulation, member checking) is needed.

Thank you for this comment. The coding procedures, including open coding, code review by a second researcher, consensus discussions, and consultation of a third researcher in case of

disagreement, were already described in the Methods section. To further improve transparency regarding reflexivity and trustworthiness, we added information on the interviewer's role in the Data Collection section and included Supplementary Text S2 describing the involvement of both an independent researcher (YK) and SANO-affiliated researchers (SGP and BW) in data interpretation, documentation of analytical decisions throughout the study, and the fact that member checking was not performed.

"The researchers involved in data analysis had different relationships to the SANO trial. YK was not involved in the SANO trial, whereas SGP and BW were members of the SANO study group. The involvement of both independent and SANO-affiliated researchers facilitated critical reflection on data interpretation and helped minimise the influence of individual perspectives.

Analytical decisions were documented throughout the study to enhance transparency and consistency. Participants did not review transcripts or study findings (member checking was not performed)."

2. Interpretation limited to descriptive aggregation

The current results largely resemble a collection of opinions rather than a structured synthesis. The manuscript would benefit from a clearer integration of findings across institutions and disciplines.
It remains unclear whether there were conflicting opinions between specialties (e.g., surgeons vs gastroenterologists) and how these differences were handled.
The authors should provide a more analytical interpretation beyond listing barriers and facilitators.

Without deeper synthesis, the study risks being perceived as a descriptive survey rather than a rigorous qualitative analysis.

Response: Thank you for this comment. Our aim was to identify implementation determinants and organise these within the CFIR framework rather than compare perceptions between professional groups. During analysis, we did not identify consistent differences between specialties. Instead, variation was observed primarily between hospitals and local organisational structures, which is reflected in several themes such as planning, multidisciplinary collaboration, and responsibility for follow-up care. To make this clearer, we added a statement to the Results section.

"Across interviews, determinants were largely consistent across professional disciplines. Where variation occurred, this was primarily related to local organisational structures and workflows rather than specialty-specific perspectives."

3. Positioning within existing literature

The discussion of similar qualitative or implementation studies is limited.

Are similar CFIR-based or qualitative studies available in this field (e.g., other cancers or surveillance strategies)?
How does this study add to existing implementation science literature?

Providing a clearer context would help readers understand whether this approach is standard and how generalisable the findings are.

Response: To further position our findings within the implementation science literature, we added a brief comparison with active surveillance in prostate cancer, where similar organisational, communicative, and patient-related determinants have been reported. We added this in the discussion section. In addition, we emphasised that our study is the first to systematically identify implementation determinants of active surveillance after nCRT for oesophageal cancer using the CFIR framework.

"Similar implementation challenges have been reported in active surveillance for prostate cancer, particularly regarding communication, care coordination, and healthcare processes"

"This is the first study to systematically identify implementation determinants of active surveillance after nCRT for oesophageal cancer using the CFIR framework, providing a foundation for targeted implementation strategies."

4. Tables are difficult to interpret

The tables (particularly Table 2) are difficult to interpret.

The meaning of symbols (e.g., - / + / -+) is not sufficiently explained.
The table structure is overly condensed and lacks clarity.
It is difficult to understand the relative importance or frequency of each theme.

The tables should be revised to improve readability and interpretability, possibly by adding explanations, examples, or quantification where appropriate.

Response: We have revised Table 2 by providing a clearer explanation of the symbols used to indicate barriers and facilitators and by improving the table formatting. We also edited the layout of Table 1 for a clearer overview.

5. Need for clearer synthesis and take-home message

The manuscript would benefit from a more structured synthesis of findings.

A conceptual model or summary figure integrating barriers and facilitators would enhance clarity.
Practical implications for clinicians and policymakers should be more explicitly stated.
Currently, the conclusions remain somewhat generic and do not fully reflect the richness of the data.

Thank you for this comment. The identified barriers and facilitators are already organised according to the CFIR domains and summarised in Table 2. To improve synthesis and practical interpretation, we strengthened the Discussion and Conclusion sections by emphasising the overarching implementation determinants and their implications for clinicians and policymakers.

"Overall, implementation challenges appeared to be driven primarily by organisational and logistical complexity rather than by consistent differences between professional groups, highlighting the importance of organisational readiness for successful implementation."

Conclusion adjusted

"We identified important implementation challenges, including logistical demands, diagnostic uncertainty, and increased workload. Our findings suggest that successful implementation of active surveillance requires organisational readiness, characterised by coordinated follow-up pathways, clear allocation of responsibilities, multidisciplinary collaboration, and specialised expertise. For policymakers, sustainable reimbursement structures, national guidance, and continued evidence generation may support broader implementation. While active surveillance was generally considered feasible, sustained evidence on its efficacy and safety remains essential for widespread adoption."

Reviewer: 2
Dr. Pooja Prasad, Royal Victoria Infirmary
Comments to the Author:
A well-written article addressing a relevant issue given the increasing focus on organ-preservation strategies in this patient population. Appropriate study question and methods to address this. Clear that capturing patient/participant experience is vital and will need to be captured at the end of the prospective study.

Response:
We thank the reviewer for the positive assessment of our manuscript and for recognising the relevance of this topic in the context of organ-preservation strategies for oesophageal cancer. We appreciate the constructive comments and suggestions, which have helped us

improve the clarity and quality of the manuscript. Detailed responses to each comment are provided below.

A few areas that will need to be addressed when revised:

1. No. of clinicians- 42 attended and 32 contributed. Are there reasons for 10 participants not providing data or for this to not be included?

Response:
Thank you for this comment. Although 42 clinicians attended the focus group interviews, 10 participants did not actively contribute to the discussions and therefore did not provide analyzable qualitative data. As our analysis was based on participants who contributed verbally during the interviews, only the 32 contributing participants were included in the study sample. We have clarified this in the Results section and the footnote of Table 2.

2. Data saturation- was this reached? Did the iterative refinement of the topic guide after each set of interviews suggest data saturation was being reached? This is important w regards to qualitative studies and should be addressed

Response: Thank you for this comment. Formal data saturation was not used to determine the sample size, as participating hospitals were selected a priori to ensure representation of different hospital types and disciplines. However, following the fourth interview, no further changes to the interview guide were required and no substantially new information emerged in the subsequent interviews. We have clarified this in the Methods section.

Following the fourth interview, no further changes to the interview guide were required and no substantially new information emerged in the subsequent interviews. Although formal data saturation was not assessed, these observations suggested that the included sample provided sufficient information to address the study aim."

3. Limitations- reflexivity ought to be discussed given it is a component of the COREQ checklist used and there is potential for bias with regards to whether limitations/barriers can be captured accurately"

Response: We agree that reflexivity is an important aspect of qualitative research and should be acknowledged as a study limitation. We have added a statement to the Limitations section discussing the potential influence of researchers' involvement in the SANO trial on data collection and interpretation, as well as the measures taken to mitigate this risk.

"Furthermore, SGP and BW were involved in the SANO trial, which may have influenced data collection and interpretation. To mitigate this risk, interviews were conducted using a predefined semi-structured interview guide with open-ended questions, and initial coding was performed by an independent researcher (YK) who was not involved in the SANO trial."

4. Financial implications- key barrier to implementation recognised and mentioned but the discussion lacks adequate exploration of this

Response: We agree that the financial implications of active surveillance warrant further discussion. We have expanded the Discussion section to emphasise that implementation of active surveillance may result in a redistribution of workload and healthcare resources across specialties, making appropriate reimbursement structures and sustainable funding mechanisms important prerequisites for broader implementation.

"However, successful implementation will also require alignment of financial incentives with the surveillance care pathway. While active surveillance may reduce surgical volume, it increases demands on gastroenterology, nuclear medicine, coordination, and long-term follow-up services. Appropriate reimbursement structures are therefore essential to ensure that implementation remains financially sustainable across specialties and healthcare organisations."

Few minor formatting / grammatical issues to address:
1. Table 2- key for facilitators/barriers symbol and needs clear formatting (+/- for example)
Response: this has been adjusted and clarified

2. Page 9 Line 26- 'practitioner' --> incorrect spelling (manuscript says practicioner)
Response: this has been adjusted.

3. reference 11- no DOI etc included. If this is unpublished data or in press, please state this explicitly (given it is the same study group and addressing the topic of EUS utility in surveillance strategies)

Response: this has been added

Reviewer: 3
Prof. Muntzer Mughal, University College London Hospitals NHS Foundation Trust
Comments to the Author:
This is an excellent study to prepare the broad healthcare ecosystem for the likely incorporation of active surveillance after neoadjuvant chemoradiotherapy for oesophageal cancer.

Response: We thank the reviewer for the positive assessment of our study and for recognising its relevance in preparing healthcare systems for the implementation of active surveillance after neoadjuvant chemoradiotherapy for oesophageal cancer. We appreciate

the constructive suggestion regarding the accessibility of the abstract and have revised the manuscript accordingly.

The abstract needs to explain the acronyms as well as terminology specific to this study: 'inner setting' and 'outer setting'. Otherwise, the purpose of the abstract is lost, considering that many readers look at the abstract to, in my opinion, justifiably decide whether to read the entire paper.

Response: Thank you for this comment. We agree that readers may be unfamiliar with CFIR-specific terminology. To improve clarity while maintaining the abstract word limit, we added brief explanations of the terms "outer setting" and "inner setting" when they are first introduced in the abstract.

“The CFIR comprised five domains: innovation, **outer setting (external healthcare environment)**, **inner setting (hospital organisational context)**, characteristics of individuals, and process of implementation.”
