# Supplementary material for: Barriers and facilitators for implementing active surveillance after neoadjuvant chemoradiotherapy for oesophageal cancer in Dutch hospitals: a qualitative study
Source: BMJ Open. 2026 Nov 11;16(11):e119825. doi: 10.1136/bmjopen-2026-119825 (PMC13756475; doi:10.1136/bmjopen-2026-119825)
Supplement: Supplementary file 2 [file bmjopen-16-11-s001.docx]

**Supplementary materials**

**Text S1 CFIR-Aligned Topic Guide for Focus Group Interviews with Healthcare Professionals**

**Aim:**
To evaluate the implementation of active surveillance within the SANO care pathway using the Consolidated Framework for Implementation Research (CFIR).

**Introduction**

Brief explanation of active surveillance as the *intervention* and short introduction to CFIR to structure the discussion.

**Intervention Characteristics (SANO Active Surveillance Care Pathway)**

**1. Perceived complexity and relative advantage**

- Which components of the SANO active surveillance care pathway do you experience as most challenging or problematic?
- What strategies have been used locally to address these challenges?
- Which components are easiest to organise or generate the least discussion within the multidisciplinary team?
- Compared with the original SANO trial, were there additional challenges during SANO-2?
- To what extent does your personal attitude toward active surveillance (e.g. enthusiasm or caution) influence its implementation?

**2. Evidence strength and quality / Design quality and packaging**

- Which diagnostic investigations are considered essential within the active surveillance protocol?
- How do you perceive the clinical value of the first clinical response evaluation (CRE-1)?
- Does combining PET-CT with gastroscopy and EUS add value?
- Can EUS be performed based on indication only? If so, which indications are appropriate?

**3. Adaptability**

- How should high-grade dysplasia detected during active surveillance be managed?
- How could this be optimally incorporated into the care pathway in the future?

**Inner Setting: Structural Characteristics and Implementation Climate**

**4. Planning, coordination, and available resources**

- Is obtaining bite-on-bite biopsies feasible in routine clinical practice?
- Is it feasible to schedule gastroscopy and PET-CT on the same day?
- Is it possible to plan CREs at the intended follow-up intervals?
- Can the diagnostic schedule be maintained after an additional clinical response evaluation?
- Is scheduling surgery within two weeks after a positive CRE feasible?

**Inner Setting: Networks and Communications / Characteristics of Individuals**

**5. Offering active surveillance to patients**

- Which professional(s) currently introduce active surveillance as a treatment option to patients?
- How does this discussion typically proceed?
  - What facilitates these discussions?
  - What barriers are encountered?
- Who should ideally introduce active surveillance as a treatment option?
- At which time point(s) in the treatment trajectory should this be discussed?
- What is the optimal timing for informed consent, and which discipline should be responsible?

**Inner and Outer Setting: Implementation Networks and External Context**

**6. Multidisciplinary collaboration and role clarity**

- Are there barriers in collaboration between the involved disciplines?
- How do you evaluate the frequency and content of multidisciplinary team meetings?
- Which discipline should act as the primary responsible clinician during active surveillance?
- Have you observed changes in workload distribution between disciplines?

**Outer Setting: External Policies, Incentives, and Patient Needs**

**7. Financial and policy context**

- Has the hospital experienced challenges related to reimbursement of active surveillance diagnostics?
  - If yes, for which services?
  - Are future reimbursement challenges anticipated?

**8. Patient needs, resources, and external stakeholder involvement**

- Has involvement of the patient advocacy group (SPKS) influenced patient treatment choices?
  - If so, in what way?

**Closure of the Focus Group**

Table S1. Members of the SANO study group, besides the authors who are indicated on the title page

| Name | Department | Centre |
| --- | --- | --- |
| Arjun D. Koch | Gastroenterology | Erasmus MC – University medical centre |
| Suzan Nikkessen | Gastroenterology | Erasmus MC – University medical centre |
| Ate van der Gaast | Medical oncology | Erasmus MC – University medical centre |
| Roelf Valkema | Nuclear medicine | Erasmus MC – University medical centre |
| Michail Doukas | Pathology | Erasmus MC – University medical centre |
| Lindsey Oudijk | Pathology | Erasmus MC – University medical centre |
| Roy S. Dwarkasing | Radiology | Erasmus MC – University medical centre |
| Joost J. Nuyttens | Radiotherapy | Erasmus MC – University medical centre |
| Berend van der Wilk | Surgery | Erasmus MC – University medical centre |
| Ben Eyck | Surgery | Erasmus MC – University medical centre |
| Bo Jan Noordman | Surgery | Erasmus MC – University medical centre |
| Maartje Valkema | Surgery | Erasmus MC – University medical centre |
| Pieter C. van der Sluis | Surgery | Erasmus MC – University medical centre |
| David van Klaveren | Public Health | Erasmus MC – University medical centre |
| Hester F. Lingsma | Public Health | Erasmus MC – University medical centre |
|  |  |  |
| Tanya M. Bisseling | Gastroenterology | Radboud University Medical Centre |
| Geert J. Bulte | Gastroenterology | Radboud University Medical Centre |
| Peter D. Siersema | Gastroenterology | Radboud University Medical Centre |
| Harm Westdorp | Medical oncology | Radboud University Medical Centre |
| Erik H. Aarntzen | Nuclear medicine | Radboud University Medical Centre |
| Chella S. van der Post | Pathology | Radboud University Medical Centre |
| Maartje C. van Rijk | Radiology | Radboud University Medical Centre |
| Pètra M. Braam | Radiotherapy | Radboud University Medical Centre |
| Heidi Rütten | Radiotherapy | Radboud University Medical Centre |
| Marcel Verheij | Radiotherapy | Radboud University Medical Centre |
| Bastiaan Klarenbeek | Surgery | Radboud University Medical Centre |
|  |  |  |
| Serge J. Zweers | Gastroenterology | Maasstad Hospital |
| Lieke Hol | Gastroenterology | Maasstad Hospital |
| I. Lisanne Holster | Gastroenterology | Maasstad Hospital |
| Ewout F.W. Courrech Staal | Radiology | Maasstad Hospital |
| Karen E. Hamoen | Pathology | Maasstad Hospital |
| Trudy Rapmund | Surgery | Maasstad Hospital |
| Erwin van der Harst | Surgery | Maasstad Hospital |
| Peter-Paul L.O. Coene | Surgery | Maasstad Hospital |
|  |  |  |
| Andries van der Linden | Gastroenterology | Ziekenhuisgroep Twente Hospital |
| Huseyin Aktas | Gastroenterology | Ziekenhuisgroep Twente Hospital |
| Polat Dura | Gastroenterology | Ziekenhuisgroep Twente Hospital |
| Ronald Hoekstra | Medical oncology | Ziekenhuisgroep Twente Hospital |
| Ali Agool | Nuclear medicine | Ziekenhuisgroep Twente Hospital |
| Joop van Baarlen | Pathology | Ziekenhuisgroep Twente Hospital |
| Ellen M. Hendriksen | Radiotherapy | Ziekenhuisgroep Twente Hospital |
| Henk Jan Mantel | Surgery | Ziekenhuisgroep Twente Hospital |
| Marc van Det | Surgery | Ziekenhuisgroep Twente Hospital |
| Ewout A. Kouwenhoven | Surgery | Ziekenhuisgroep Twente Hospital |
|  |  |  |
| Rutger Quispel | Gastroenterology | Reinier de Graaf Gasthuis |
| Sana A. Mulder | Gastroenterology | Reinier de Graaf Gasthuis |
| Arjan J. Verschoor | Medical oncology | Reinier de Graaf Gasthuis |
| Marc R.J. ten Broek | Nuclear medicine | Reinier de Graaf Gasthuis |
| René J. Dallinga | Radiology | Reinier de Graaf Gasthuis |
| Karen J. Neelis | Radiotherapy | Reinier de Graaf Gasthuis |
| Erlinde de Graaf | Surgery | Reinier de Graaf Gasthuis |
| Stijn van Esser | Surgery | Reinier de Graaf Gasthuis |
|  |  |  |
| Jolanda M. van Dieren | Gastroenterology | The Netherlands Cancer Institute |
| Thomas R. de Wijkerslooth | Gastroenterology | The Netherlands Cancer Institute |
| Marieke A. Vollebergh | Medical oncology | The Netherlands Cancer Institute |
| Emilia C. Owers | Nuclear medicine | The Netherlands Cancer Institute |
| Annemarieke Bartels-Rutten | Radiology | The Netherlands Cancer Institute |
| Liudmila L. Kodach | Pathology | The Netherlands Cancer Institute |
| Francine E.M. Voncken | Radiotherapy | The Netherlands Cancer Institute |
| Yvonne Hilhorst | Surgery and gastroenterology | The Netherlands Cancer Institute |
| Marjolein Warmerdam | Surgery and gastroenterology | The Netherlands Cancer Institute |
|  |  |  |
| Sietske Corporaal | Gastroenterology | Frisius Medical Centre |
| Edward Fiets | Medical oncology | Frisius Medical Centre |
| Marco B. Polée | Medical oncology | Frisius Medical Centre |
| Anne Marij G. van Burg | Nuclear medicine | Frisius Medical Centre |
| Judith Nieken | Pathology | Frisius Medical Centre |
| Rinze Wolf | Radiology | Frisius Medical Centre |
| Vera Oppedijk | Radiotherapy | Frisius Medical Centre |
| Marloes Emous | Surgery | Frisius Medical Centre |
| Daniel A. Hess | Surgery | Frisius Medical Centre |
|  |  |  |
| Eva Kouw | Gastroenterology | Gelre Hospital |
| Willemien Erkelens | Gastroenterology | Gelre Hospital |
| S. Cathrien S. Tromp – van Driel | Medical oncology | Gelre Hospital |
| Marc D. Zuijdwijk | Nuclear medicine | Gelre Hospital |
| H. Doornewaard | Pathology | Gelre Hospital |
| Karin Muller | Radiotherapy | Gelre Hospital, Radiotherapiegroep Deventer |
| Peter van Duijvendijk | Surgery | Gelre Hospital |
| Eelco B. Wassenaar | Surgery | Gelre Hospital |
| Edwin S. van der Zaag | Surgery | Gelre Hospital |
|  |  |  |
| Wouter L. Curvers | Gastroenterology | Catharina Hospital |
| Geert-Jan Creemers | Medical oncology | Catharina Hospital |
| Mark J. Roef | Nuclear medicine | Catharina Hospital |
| Ineke van Lijnschoten | Pathology | Catharina Hospital |
| Joost Nederend | Radiology | Catharina Hospital |
| Maurice J.C. van der Sangen | Radiotherapy | Catharina Hospital |
| Tom C.G. Budiharto | Radiotherapy | Catharina Hospital |
| Fanny F.B.M. Heesakkers | Surgery | Catharina Hospital |
| Misha D. Luyer | Surgery | Catharina Hospital |
|  |  |  |
| Liekele E. Oostenbrug | Gastroenterology | Zuyderland Medical Centre |
| Fabienne A.R.M. Warmerdam | Medical oncology | Zuyderland Medical Centre |
| Wendy Schreurs | Nuclear medicine | Zuyderland Medical Centre |
| Robert Riedl | Pathology | Zuyderland Medical Centre |
| Roy F.A. Vliegen | Radiology | Zuyderland Medical Centre |
| Jeroen Buijsen | Radiotherapy | Zuyderland Medical Centre |
| Ilse Stohr | Surgery | Zuyderland Medical Centre |
| Eric H.J. Belgers | Surgery | Zuyderland Medical Centre |
| Meindert N. Sosef | Surgery | Zuyderland Medical Centre |
|  |  |  |
| Jolein van der Kraan | Gastroenterology | Leiden University Medical Centre |
| Marije Slingerland | Medical oncology | Leiden University Medical Centre |
| Richard Raghoo | Nuclear medicine | Leiden University Medical Centre |
| A. Stijn L.P. Crobach | Pathology | Leiden University Medical Centre |
| Aart J. van der Molen | Radiology | Leiden University Medical Centre |
| Susan J.C.L.M. Quix | Surgery | Leiden University Medical Centre |
| Wobbe O. de Steur | Surgery | Leiden University Medical Centre |
| Henk H. Hartgrink | Surgery | Leiden University Medical Centre |
|  |  |  |
| Wouter L. Hazen | Gastroenterology | Elisabeth Tweesteden Hospital |
| Laurens V. Beerepoot | Medical oncology | Elisabeth Tweesteden Hospital |
| David E. Ploeg | Pathology | Elisabeth Tweesteden Hospital |
| Tom Rozema | Radiotherapy | Elisabeth Tweesteden Hospital |
| Ilse A.C. Vermeltfoort | Nuclear medicine | Elisabeth Tweesteden Hospital |
| Walther Jansen | Surgery | Elisabeth Tweesteden Hospital |
